# “Fake it till You Make it”! Contaminating Rubber Hands (“Multisensory Stimulation Therapy”) to Treat Obsessive-Compulsive Disorder

**DOI:** 10.3389/fnhum.2019.00414

**Published:** 2020-01-09

**Authors:** Baland Jalal, Richard J. McNally, Jason A. Elias, Sriramya Potluri, Vilayanur S. Ramachandran

**Affiliations:** ^1^Department of Psychiatry, Behavioural and Clinical Neuroscience Institute, University of Cambridge School of Clinical Medicine, Cambridge, United Kingdom; ^2^Department of Psychology, Harvard University, Cambridge, MA, United States; ^3^Obsessive-Compulsive Disorder Institute, McLean Hospital, Belmont, MA, United States; ^4^Department of Psychiatry, Harvard Medical School, Boston, MA, United States; ^5^Center for Brain and Cognition, University of California, San Diego, San Diego, CA, United States

**Keywords:** obsessive-compulsive disorder (OCD), rubber hand illusion, therapy, contamination fears, exposure and response prevention (ERP), multisensory integration

## Abstract

Obsessive-compulsive disorder (OCD) is a deeply enigmatic psychiatric condition associated with immense suffering worldwide. Efficacious therapies for OCD, like exposure and response prevention (ERP), are sometimes poorly tolerated by patients. As many as 25% of patients refuse to initiate ERP mainly because they are too anxious to follow exposure procedures. Accordingly, we proposed a simple and tolerable (immersive yet indirect) low-cost technique for treating OCD that we call “multisensory stimulation therapy.” This method involves contaminating a rubber hand during the so-called “rubber hand illusion” (RHI) in which tactile sensations may be perceived as arising from a fake hand. Notably, Jalal et al. (2015) showed that such fake hand contamination during the RHI provokes powerful disgust reactions in healthy volunteers. In the current study, we explored the therapeutic potential of this novel approach. OCD patients (*n* = 29) watched as their hidden real hand was being stroked together with a visible fake hand; either synchronously (inducing the RHI; i.e., the experimental condition; *n* = 16) or asynchronously (i.e., the control condition; *n* = 13). After 5 min of tactile stimulation, the rubber hand was contaminated with fake feces, simulating conventional exposure therapy. Intriguingly, results suggested sensory assimilation of contamination sensations into the body image *via* the RHI: patients undergoing synchronous stimulation did not report greater contamination sensations when the fake hand was initially contaminated relative to asynchronous stroking. But contrary to expectations, they did so after the rubber hand had been contaminated for 5 min, as assessed *via* disgust facial expressions (a secondary outcome) and *in vivo* exposure (upon discontinuing the illusion). Further, to our surprise, synchronous and asynchronous stroking induced an equally vivid and fast-emerging illusion, which helps explain why both conditions initially (5 min after initiating tactile stimulation) provoked contamination reactions of equal magnitude. This study is the first to suggest heightened malleability of body image in OCD. Importantly, it may pave the way for a tolerable technique for the treatment of OCD—highly suitable for poorly resourced and emergency settings, including low-income and developing countries with minimal access to high-tech solutions like virtual reality.

## Introduction

Obsessive-compulsive disorder (OCD) is a deeply enigmatic psychiatric condition that afflicts 2% to 3% of the general population (Robins et al., 1984; Ruscio et al., 2010). One variant of OCD is characterized by severe contamination fears and excessive cleansing rituals (Rachman, 2004; Markarian et al., 2010). These patients may feel anxious even after incidents of slight “contamination” (e.g., touching a door knob) and might spend hours painstakingly washing and scrubbing their hands—sometimes until they bleed. The primary treatment for OCD is called exposure and response prevention (ERP; Meyer, 1966). During ERP, the patient is first “contaminated” (e.g., touches a toilet bowl), which can trigger an acute spike in anxiety, and then prevented from performing the compulsive ritual (e.g., washing hands). This procedure may help the patient experience a subsequent decrease in anxiety, resulting in habituation (Abramowitz et al., 2009). But unsurprisingly, many OCD patients do not benefit from ERP (Kozak, 1999); the notion of being contaminated in this crude fashion is simply too unbearable. Alarmingly, 50% of patients who start ERP do not improve, 20% drop out prematurely, and 25% refuse to initiate therapy (Kozak, 1999; Schruers et al., 2005; Abramowitz, 2006), mainly due to fear of treatment (Maltby and Tolin, 2005). As such, developing gentler (less distressing) interventions for OCD represents an unmet need.

To overcome challenges of existing exposure therapies, we recently proposed a simple and tolerable (immersive yet indirect) low-cost technique for the treatment of OCD (Jalal et al., 2015) that we call “multisensory stimulation therapy.” Healthy volunteers watched as their occluded real hand was being stroked together with a visible fake hand in precise synchrony, producing the so-called “rubber hand illusion” (RHI; Botvinick and Cohen, 1998). After 5 min of such tactile stimulation, we contaminated the dummy with fake feces, in effect, mimicking traditional exposure therapy. To our astonishment, participants reported disgust sensations—as if arising from the rubber hand! This finding with potential clinical utility (discussed in more detail below) has since been replicated in a large Japanese sample, suggesting the effect is both robust and cross-culturally reliable (Nitta et al., 2018).

One interpretation for the emergence of the RHI evokes the “Bayesian logic” of perceptual systems (e.g., Armel and Ramachandran, 2003; Ramachandran et al., 2011; Jalal et al., 2015). The brain’s sensory system is hardwired to detect statistical correlations that provide the basis for making predictions and, ultimately, visual representations of the external world, including one’s body (see also Corlett et al., 2011). The brain considers it highly unlikely that the random stroking *seen* on the fake hand and *felt* on the real hand is due simply to chance; it infers therefore that the sensations must be arising from the rubber hand, however absurd. As such, the illusion is driven by bottom-up mechanisms (i.e., statistical correlations between senses) and any object in theory could become part of one’s body image including a table (Armel and Ramachandran, 2003). Consistent with this account, the RHI does not occur (or is greatly diminished) following asynchronous stimulation of the real and rubber hand. This “gold standard” control procedure shows the importance of spatial and temporal congruence of the tactile and visual inputs in driving the illusion (e.g., Shimada et al., 2009).

To date, research has explored various measures and versions of the RHI (e.g., Armel and Ramachandran, 2003; Costantini and Haggard, 2007; Ehrsson et al., 2007; Capelari et al., 2009; Kammers et al., 2009; Ramachandran et al., 2011). The basic effect emerges fairly quickly, in most healthy volunteers usually around 10–30 s after the synchronized stroking begins (Ehrsson, 2012). In our own studies, we have found that the illusion is reliably induced in healthy individuals within 2.5–5 min of tactile stimulation (e.g., in approximately 73% of subjects across two separate experiments; see Jalal et al., 2015; see also Armel and Ramachandran, 2003). The illusion is most commonly assessed with a subjective measure of limb ownership and an objective test of proprioceptive drift, where participants after the illusion onset close their eyes and point to the direction of their real hand. Botvinick and Cohen (1998) showed that after RHI induction, participants point to the artificial hand instead of their real hand unlike in the asynchronous control condition, and that the degree of this displacement is associated with the prevalence of the RHI over time (i.e., as measured within a 30-min stimulation period). In line with this, Tsakiris and Haggard (2005) demonstrated that continuous tactile stimulation during the RHI gradually increases such proprioceptive drift, suggesting a gradual intensifying of the illusion over time. This proprioceptive drift test correlates with the subjective vividness of the illusion (e.g., Longo et al., 2008).

The RHI has also been examined in psychiatric groups: for example, one study found a stronger illusion and faster onset in schizophrenia, suggesting a malleable self-representation in this population (Peled et al., 2000). Comparable results were reported in patients with eating disorders, who likewise have a pronounced RHI compared to healthy volunteers (Eshkevari et al., 2012). Other studies have revealed a more complex picture vis-à-vis body-related processing in psychopathology. For instance, although patients with posttraumatic stress disorder (PTSD; i.e., with dissociative symptoms) initially have a more intense illusion than do healthy controls, after three consecutive trials (over the course of 2 weeks), a comparable intensity to that of healthy subjects was reported (Lev-Ari and Hirschmann, 2016). Kaplan et al. (2014) did not find the intensity of the RHI to differ in patients with body dysmorphic disorder (BDD) and healthy controls, yet surprisingly, the BDD group displayed proprioceptive drift towards the rubber hand in both the synchronous and asynchronous control condition, unlike healthy individuals, who only did so in the RHI condition as expected. Finally, children with autism spectrum disorders (ASD) have a delayed susceptibility to the illusion (i.e., exhibit a later illusion onset compared to non-autistic children). Notably, children with ASD who have lower levels of empathy are less likely to experience the RHI (Cascio et al., 2012). Taken together, these studies suggest that some forms of psychopathology are associated with aberrant self-referential processing as assessed on the RHI.

To date, no studies have examined the RHI in OCD. The illusion may be particularly pertinent to OCD given the role of dopamine in the pathophysiology of the disorder (e.g., Denys et al., 2004; Koo et al., 2010). Although the function of dopamine in OCD is multifaceted (e.g., Fineberg et al., 2007), research has shown that dopamine antagonists [as an adjunct to selective serotonin reuptake inhibitor (SSRI) drugs] can reduce OCD symptoms (i.e., augment the effects of SSRIs; Vulink et al., 2009). In contrast, dopamine agonists can generate OCD-like behaviors in animals (Szechtman et al., 1998) and humans (Borcherding et al., 1990), providing clues about the functional role of dopamine in OCD.

Interestingly, research suggests that dopamine is a key modulator of multisensory integration as assessed *via* the RHI. For instance, the dopamine releaser drugs ketamine and dexamphetamine (with potential to trigger schizophrenia-like symptoms; Angrist and Gershon, 1970; Pomarol-Clotet et al., 2006) augment the illusion during regular synchronous stroking, but curiously also, in the (illusion-attenuating) asynchronous control condition (Albrecht et al., 2011; Morgan et al., 2011). Analogously, patients with Parkinson’s disease (receiving dopaminergic drugs) fail to reject the RHI in the asynchronous condition as strongly as healthy control participants do, according to the authors, possibly due to dopamine dysregulation (Ding et al., 2017). Collectively, this research is in keeping with findings that schizophrenia (a disorder of dopamine abnormality; e.g., Howes et al., 2015) results in heightened illusory effects, and points to the pervasive role of dopamine in self-referential processing.

Research should disclose whether OCD is associated with multisensory processing abnormalities. By beginning to probe the corporeal self in OCD, one may eventually clarify how the processes that produce a sense of body ownership differ in this disorder vs. other psychiatric conditions. Indeed, if research reveals aberrant somatosensory integration in OCD, efforts to establish specificity could elucidate OCD etiology and differentially inform novel treatments (e.g., drug and behavioral interventions) aiming at restoring aspects of self-referential processing (also see Eshkevari et al., 2012).

The illusion may be of special interest to contamination-related OCD, i.e., provide an experimental probe for exploring pathological disgust and novel therapeutic techniques. As noted, we have shown that contaminating the fake hand during the RHI provokes OCD-like disgust reactions in healthy volunteers (Jalal et al., 2015): in this study, 81% of participants reported greater disgust during synchronous stroking vs. the asynchronous control condition, and, on overage, those undergoing the RHI reported significantly higher levels of disgust. In a “direct replication study,” Nitta et al. (2018) likewise showed that such “exposure” during the RHI triggered greater disgust reactions than asynchronous stroking in healthy individuals from Japan.

Notably, disgust plays a key role in OCD and is a strong predictor of contamination fears (e.g., Olatunji et al., 2005; see also Deacon and Olatunji, 2007; Olatunji et al., 2007; for reviews, see Ludvik et al., 2015; Knowles et al., 2018). Although disgust and contamination aversion overlap, they are indeed distinct concepts. Disgust is a basic emotion that induces a unique response (e.g., a facial expression; Rozin and Fallon, 1987), whereas contamination fears arise from *post hoc* interpretive processes, e.g., triggered by disgust or related emotions like anxiety (Rachman, 2004; also see Ludvik et al., 2015). Like disgust, anxiety is an independent driver of contamination fears (but may interact with disgust to trigger contamination concerns; Cisler et al., 2007). Interestingly, although traditional ERP triggers and degrades anxiety and washing urges (Rachman, 2004; Cougle et al., 2007), research suggests that disgust is also amenable to exposure therapy in OCD (McKay, 2006).

Although the results of Jalal et al. (2015) comport with the literature on ERP (i.e., disgust induced by “fake hand exposure” mirrors the effects of *in vivo* exposure; e.g., McKay, 2006), several issues remain vis-à-vis the clinical utility of this RHI contamination procedure. First, research should extend this work to a clinical population to assess the therapeutic use of the RHI; i.e., it is important to establish the presence of this basic “RHI contamination effect” in OCD patients. Second, to the extent that such rubber hand exposure evokes clinically relevant contamination reactions in OCD, research should examine whether this eventually leads to habituation.

Such research may have important treatment implications: if contaminating a fake hand during the RHI provokes contamination reactions (akin to ERP) *via* an immersive multisensory mechanism, this may pave the way for a novel (tolerable) intervention. As noted, such dummy contamination may eventually (after an extended period and/or repeated trials) lead to habituation, i.e., overall global reduction in contamination fears, analogous to conventional ERP. Another possibility is that contaminating a fake hand during the RHI, minimally, is useful during the initial stages of ERP (e.g., in an “exposure hierarchy”; Wolpe, 1958; see also Abramowitz et al., 2003). This technique might sufficiently desensitize patients such that they are willing to undertake ERP, providing a convenient “transitional link” (Jalal et al., 2015).

### Primary Study Aims

In the current study, the key aim was to explore the therapeutic potential of the RHI for OCD. We examined whether “contaminating” the rubber hand during the illusion would result in greater contamination sensations as compared to the asynchronous control condition. We also tested whether such dummy contamination eventually resulted in habituation, assessed both during the illusion and during an *in vivo* exposure procedure immediately upon discontinuing the illusion (i.e., ceasing the stimulation of the real and rubber hand).

#### Hypotheses

If contaminating the fake hand during the RHI (5 min after initiating stroking) provokes greater disgust than asynchronous stroking in healthy individuals (Jalal et al., 2015; Nitta et al., 2018)—given the role of disgust in OCD—this should also hold for patients with contamination obsessions. Moreover, considering that ERP targets both anxiety and washing urges (Rachman, 2004), RHI exposure should likewise evoke such contamination sensations overall (i.e., in addition to disgust). Finally, given that OCD patients dependably experience habituation following prolonged exposure to “contaminants” during ERP (on habituation see, e.g., Foa et al., 1983; Rachman, 2004; Abramowitz, 2006), RHI exposure should after an extended period lead to habituation [This latter hypothesis is partly grounded in research showing that the RHI emerges quickly and does not wane with time (e.g., Tsakiris and Haggard, 2005; Ehrsson, 2012), preserving the realistic nature of the exposure procedure].

Assuming that: (1) contaminating the fake hand during the RHI results in greater contamination sensations than does asynchronous stroking in OCD; and that (2) such exposure over time leads to habituation, we advanced the following hypotheses.

RHI contamination: OCD patients in the RHI condition would report greater contamination sensations (disgust, anxiety, and handwashing urges), and be more likely to exhibit a disgust facial expression, when the fake hand is contaminated (i.e., 5 min upon initiating the real and rubber hand stroking), compared to those in the asynchronous control condition.

RHI habituation: OCD patients in the RHI condition would report lower contamination sensations (disgust, anxiety, and handwashing urges), and be less likely to exhibit a disgust facial expression, 5 min after contaminating the dummy (i.e., 10 min upon initiating the real and rubber hand stroking), compared to those in the asynchronous condition.

*In vivo* exposure (habituation assessment): OCD patients in the RHI condition would report lower contamination sensations (disgust, anxiety, and handwashing urges) when their real hand is contaminated (i.e., immediately upon ceasing the stimulation of the real and rubber hand) compared to those in the asynchronous condition.

### Secondary (Exploratory) Aims

A secondary aim was to broadly explore multisensory processing in OCD. In view of research: (1) indicating that dopamine, implicated in OCD (e.g., Denys et al., 2004; Koo et al., 2010), is a modulator of multisensory processing (e.g., Albrecht et al., 2011; Morgan et al., 2011); and (2) suggesting aberrant somatosensory integration in psychiatric disorders more generally (see above), we tentatively hypothesized that OCD would be associated with atypical multisensory processing. For example, OCD patients would show high susceptibility to the illusion (indexed by illusion onset and intensity measures) compared to healthy populations (e.g., as reported in our own studies; Jalal et al., 2015). Given the exploratory (open-ended) nature of this inquiry, no directional hypothesis was made *a priori*.

## Materials and Methods

### Participant Selection and Clinical Characteristics

Study participants included 29 OCD patients recruited from the McLean Hospital Obsessive-Compulsive Disorder Institute (OCDI), an intensive residential treatment (IRT) program affiliated with Harvard Medical School. At the OCDI, patients receive intensive (2–4 h daily) cognitive–behavioral therapy and psychopharmacological management, i.e., by a team of behavioral and family therapists, psychiatrists, etc. Medications are used on a case-to-case basis (i.e., determined during weekly psychiatric assessment) and often include SSRIs (e.g., venlafaxine and clomipramine) and antipsychotics (i.e., as an adjunct to SSRIs). Although treatment duration is based on individual need, patients on average remain at the OCDI for 45 days, with 25% of patients for at least 12 weeks (Athey et al., 2015). Inclusion criteria for admission to the OCDI include major OCD-related functional impairment and lack of response to treatment in other settings. The program does not have official exclusion criteria, but patients are not admitted if they have a condition that would interfere with treatment; e.g., severe intellectual disability (mental retardation or neurodevelopmental disorders etc.), current substance abuse and active psychosis (for details on McLean Hospital’s IRT program, see also Stewart et al., 2005).

In the current study, all participants were diagnosed with OCD by an expert clinician on staff as part of standard clinical procedures based on DSM-IV or DSM-5 criteria and had disgust- and/or contamination-related obsessions. The presence of disgust- and contamination-related symptoms were defined by elevated scores on the Disgust Propensity and Sensitivity Scale-Revised (DPSS-R; van Overveld et al., 2006) and endorsement of contamination obsessions on the Dimensional Obsessive–Compulsive Scale (DOCS; Abramowitz et al., 2010; completed as part of an admission’s battery of questionnaires). This clinical assessment was not based on a specific cutoff score but whether such symptoms were present [i.e., akin to the Yale-Brown Obsessive-Compulsive Scale (Y-BOCS) symptom checklist; Goodman et al., 1989]. As the main aim of this study was to explore a novel clinical approach, no strict selection criteria were applied (aside from the general OCDI selection criteria, noted above), ensuring that our sample was representative of this patient population. As such, medicated patients were not excluded. Given all patients were undergoing IRT, they were only selected for participation insofar that it would not interfere with their treatment.

Information regarding comorbid psychiatric diagnoses was available for 27 patients [i.e., out of 29; two patients did not complete an elaborate semi-structured diagnostic interview and/or a clinician administered intake interview to determine co-occurring conditions, due to logistic reasons (e.g., unavailability of clinical staff to conduct such interviews) or interference of OCD symptoms etc.]. Of these 27 patients, 92.6% (25/27) had OCD as a primary diagnosis and 3.7% (1/27) had OCD as a secondary diagnosis (data regarding whether OCD or a related mood disorder was primary was unavailable for one patient). Individuals who did not have a primary diagnosis of OCD were diagnosed with an obsessive-compulsive-related disorder (e.g., BDD: 3.7%; 1/27) or a related mood disorder (e.g., bipolar disorder I: 3.7%; 1/27).

Moreover, 74.1% (20/27) of participants had at least one comorbid axis I diagnosis. Frequencies of most co-occurring disorders were major depressive disorder (29.6%; 8/27), dysthymic disorder/persistent depressive disorder (18.5%; 5/27), post-traumatic stress disorder (18.5%; 5/27), and generalized anxiety disorder (14.8%; 4/27), followed by eating disorder NOS/other specified feeding or eating disorder (11.1%; 3/27), specific phobia (11.1%; 3/27), excoriation/skin-picking disorder (7.4%; 2/27), panic disorder (7.4%; 2/27), hoarding disorder (7.4%/ 2/27), bulimia nervosa (3.7%; 1/27), illness anxiety disorder (3.7%; 1/27), BDD (3.7%; 1/27), depressive disorder NOS (3.7%; 1/27), and trichotillomania (3.7%; 1/27). Participants’ past diagnoses (i.e., prior to attending the OCDI), included (but were not restricted to) alcohol abuse, eating disorder NOS, major depressive disorder, specific phobia, anorexia nervosa, excoriation/skin-picking disorder, stimulant use disorder, etc. Finally, for these 27 patients for which comorbidity information was available, no patient endorsed autism spectrum disorder (i.e., on a self-reported diagnosis checklist).

Participation was restricted to those aged between 18 and 65 years old (*M* = 26.93, *SD* = 6.74, range = 18–43), and who were proficient in English. Seventy-six percent (22/29) of participants were female and 21% (6/29) were male (one participant did not provide consent for their demographic data to be shown).

### Procedure

Harvard University’s Committee on the Use of Human Subjects approved the study protocol and McLean Hospital’s Institutional Review Board formally ceded review to Harvard’s committee. Participants gave written informed consent prior to initiation of any study procedure and received monetary compensation ($20) for their time.

The participant sat behind a desk with both hands resting on it. A vertical cardboard barrier (sagittal partition) was placed on the table, just to the left of the participant’s right hand, occluding his view of his right hand. A rubber hand was placed on the left side of the cardboard. A sheet of cloth was wrapped around the wrist of the dummy extending up to the shoulder of the right arm. This arrangement prevented the participant from viewing his right hand, giving the illusion that the fake hand was his real right hand. The rubber hand was positioned in parallel to (i.e., mirrored) the real left hand. The palm of the left hand was facing down, and the left arm was positioned in an approximately 90° angle along the body with the elbow near the torso and the forearm resting on the table. The participant’s right arm was slightly extended, with the elbow slightly away from the torso and shoulder raised, allowing for the proper placement of the partition, i.e., extending from the right collarbone onto the desk. The real right forearm and hand (palm down) likewise rested on the desk, as noted, completely out of sight during the entire stimulation period.

Next, the participant was instructed to indicate orally when he or she experienced touch sensations coming from the rubber hand (this onset rating was only reported if the participant felt the illusion; the participant was not further asked about the illusion onset). The experimenter then began to stroke the participant’s right hand (i.e., dorsum, with slight fluctuation in speed and directionality) with a paintbrush while simultaneously and synchronously stroking the rubber hand with another paintbrush continuously for 10 min (i.e., without interruption to sustain the illusion). The simultaneous stroking of the rubber hand and the real right hand produces the illusion (to the participant) that the rubber one feels like his own right hand. After 5 min of such stroking, the experimenter asked the participant to rate how much the rubber hand felt like his own hand on a 20-point Likert scale (this was the only time point at which the illusion intensity was assessed). Next, the experimenter used a tissue to smear the disgust stimulus (fake feces) on the rubber hand while simultaneously dabbing a damp paper towel from a nearby water bowl on the participant’s real right hand. The damp towel placed on the occluded right hand served the purpose of mimicking the sensation of having the contaminant smeared on the participant’s real hand (see also Jalal et al., 2015). Immediately thereafter, the participant was asked to provide subjective contamination ratings (i.e., disgust, anxiety, and handwashing urge levels), and the experimenter rated the participant’s facial expression of disgust (either present or not). The tissue that had been used to “contaminate” the rubber hand and the clean paper towel was then removed from the fake and real hand; the fake feces remained on the rubber hand. The rubber hand and the participant’s real hand continued to be stroked for an additional 5 min, after which the participant again provided contamination ratings and the experimenter rated his facial expression. The stroking of the rubber hand and real hand then stopped (i.e., 10 min of uninterrupted rubber hand and real hand stimulation had elapsed). Immediately thereafter, the experimenter told the participant that he would place the disgust stimulus (referred to as the “object”) on his right hand and, accordingly, took a piece of the disgust stimulus and put it on the participant’s real right hand. At this point, the participant provided a final set of contamination ratings.

**Figure 1:**
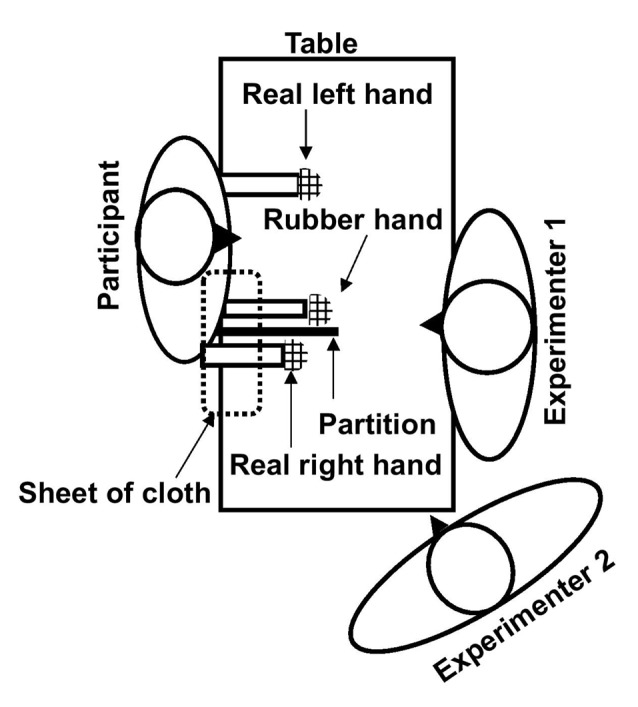
The setup of the rubber hand illusion (RHI). The role of experimenter 1 was to continuously stroke the real and rubber hand during the stimulation period and that of experimenter 2 was to obtain ratings and conduct the remaining experimental procedures (contamination procedures, etc.).

A second group of patients underwent the same procedure except that the stimulation of the rubber hand and real right hand was asynchronous (i.e., the stroking was temporally and spatially incongruent), thereby either greatly diminishing or preventing the illusion from developing (The setup of the experiment is shown in Figure 1).

### Materials and Measures

#### Yale-Brown Obsessive-Compulsive Scale (Y-BOCS)

The Y-BOCS (Goodman et al., 1989) is widely considered the “gold standard” measure for assessing OCD symptomatology in clinical research. The Y-BOCS indexes severity of obsessions and compulsions in the past week. Scores are generated from a total of 10 items, each rated on a five-point Likert scale, and scores range from 0 to 40. In the present study, patients completed the self-report version of the Y-BOCS (Steketee et al., 1996).

#### Disgust Stimulus

**Figure 2:**
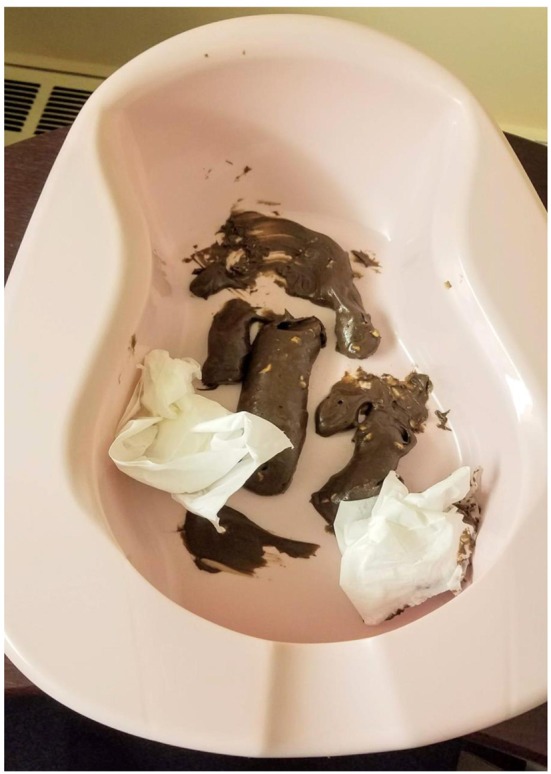
Disgust stimulus.

The disgust stimulus visually resembled and smelled of genuine feces. It consisted of food items (a mixture of chocolate and peanut butter) and was sprayed with a joke-shop odor, and placed in a bedpan. Participants were told before the study began that the stimulus was not genuine feces (Figure 2).

#### Multisensory Integration

RHI onset and intensity: the time onset of the RHI (i.e., how soon after the stroking was initiated participants felt the presence of the illusion, if at all) constituted a measure of multisensory integration. Participants were asked to indicate verbally if and when they experienced touch sensations coming from the rubber hand.

The perceived intensity of the illusion provided another measure of multisensory processing (i.e., limb ownership). Participants were asked to rate how much the rubber hand felt like their own hand (5 min after initiating the stroking), on a 20-point Likert scale, ranging from 1 (“not at all”) to 20 (“exactly like my own hand”). A more rapid onset (measured in seconds) and higher intensity rating indicated greater susceptibility to the illusion.

#### RHI Contamination

Participants were asked to provide ratings of contamination sensations (i.e., their level of disgust, anxiety, and handwashing urges), when the rubber hand was first contaminated (i.e., 5 min after initiating the stroking), on a 10-point Likert scale ranging from 1 (“not at all”) to 10 (“extremely”). Higher ratings indicated greater assimilation of contamination sensations into their body image *via* the RHI.

#### RHI Habituation

Participants were asked to provide contamination ratings (i.e., disgust, anxiety, and handwashing urge levels), 5 min after the dummy contamination procedure (i.e., 10 min after initiating the stroking), on a 10-point Likert scale ranging from 1 (“not at all”) to 10 (“extremely”). Lower ratings indicated greater habituation.

Disgust facial expressions: to further gauge participants’ disgust reactions, we observed and noted whether their facial expression indicated disgust (or not) when: (1) the rubber hand was initially contaminated; and (2) when *RHI habituation* assessment took place (i.e., 5 min after the dummy contamination).

#### *In vivo* Exposure Habituation

**Figure 3:**
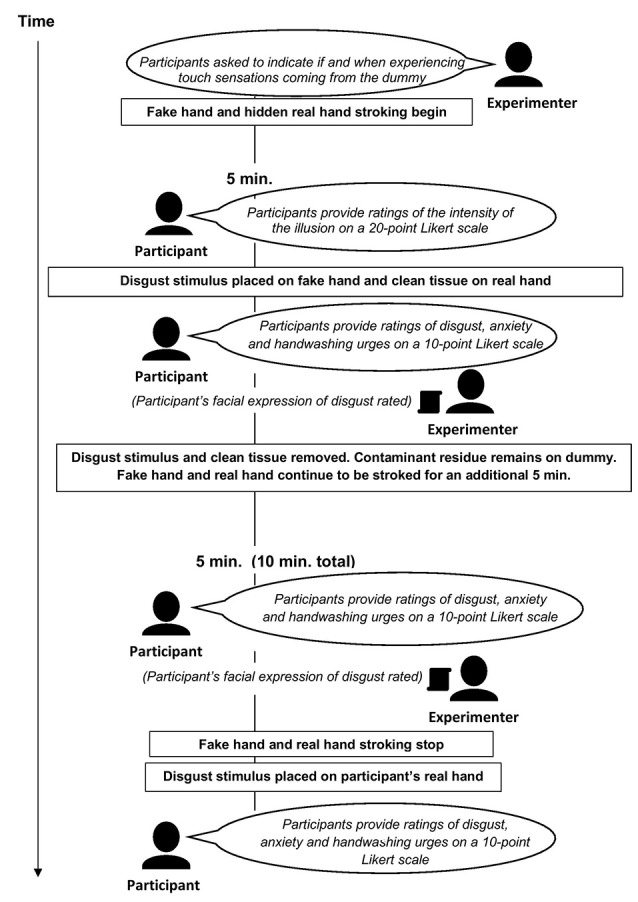
Overview of study.

Participants were asked to provide contamination ratings (i.e., disgust, anxiety and handwashing urge levels), when the experimenter contaminated the participant’s real hand (i.e., immediately after *RHI habituation* ratings were obtained), on a 10-point Likert scale ranging from 1 (“not at all”) to 10 (“extremely”). Lower ratings indicated greater habituation, an overview of the experimental procedures is shown in Figure 3.

### Statistical Analyses

The study included a quantitative between-subject cross-sectional design comparing two conditions (experimental vs. control) on the following measures: RHI contamination sensations, RHI habituation, *in vivo* exposure habituation, and multisensory integration, focusing on the between-subject effects. The study targeted the following primary outcome variables: self-reported ratings of disgust, anxiety, and handwashing urges (assessment of RHI contamination sensations and habituation effects), and RHI onset and intensity (assessment of multisensory integration). Participants’ facial expression of disgust (i.e., present or non-present; rated by the experimenter) constituted a secondary outcome measure of RHI contamination sensations and habituation.

RHI onset and intensity-dependent variables were analyzed *via* one-way ANOVA. Disgust, anxiety, and handwashing urge rating dependent variables were analyzed using a one-way MANOVA test, followed up with ANOVA *post hoc* tests. A chi-squared test was used to analyze disgust facial expression dependent variables.

For all analyses testing *a priori* hypotheses, we applied the Benjamini and Hochberg (1995) false discovery rate (FDR; e.g., McDonald, 2014) to control for potential Type I errors. Congruent with related studies (e.g., Skandali et al., 2018) and general guidelines (e.g., Genovese et al., 2002), the FDR was set at *q* < 0.15. In the current study, the Benjamini–Hochberg corrected significance level was 0.06. *P*-values shown in the text are uncorrected (i.e., raw; e.g., McDonald, 2014). Exploratory analyses and *post hoc* tests (i.e., following a significant omnibus MANOVA) were not adjusted for multiple comparisons. Multiplicity correction is not required when analyses are labeled exploratory (Bender and Lange, 2001).

For all dependent variables, the distribution of residuals was checked with Q–Q plots and the Shapiro–Wilk test; residuals were often found to depart from normality. Such variables were transformed with a log_10_(*x* + 1) and a square-root transformation to test whether these improved matters (Myers and Well, 2003). As the *F*-test is robust to minor normality departures (Blanca et al., 2017), we report untransformed data (except when otherwise specified in the text; on all figures, error bars denote standard error of the mean).

## Results

Twenty-nine OCD patients completed the study. Of these, 16 were assigned to the experimental condition (i.e., to undergo the RHI) and 13 were assigned to the control (i.e., to undergo asynchronous stroking of the real and rubber hand). One OCD patient failed to provide consent for their demographic and Y-BOCS data to be used; these were thus excluded. The final sample sizes were experimental condition *n* = 16 and control condition *n* = 13.

Additional data were missing for a few measures. Three participants did not provide an illusion time onset. One participant’s data were excluded from the “RHI contamination and habituation” analyses due to an experimental error. Likewise, a participant was excluded from these analyses for not exhibiting an adequate contamination fear response throughout the experiment [e.g., with average contamination ratings as low as 1.3 out of 10 in intensity when directly exposed to the disgust stimulus during *in vivo* exposure; for a third participant, the tissues used to stimulate the real hand and contaminate the dummy were not removed after this experimental procedure. As this protocol deviation was trivial (i.e., unlikely to impact contamination sensations), the data were not excluded. As a precaution, the data were also analyzed while excluding this participant; the results remained unaltered]. For demographic and clinical characteristics of participants, see Table 1.

**Table 1:** Demographic and Clinical Characteristics of Participants^a^.

Condition	Experimental † (*n* = 15)		Control (*n* = 13)		Comparison
	*M*	(*SD*)	*M*	(*SD)*)	*F*_df_
Age	26.60	(7.32)	27.31	(6.28)	*F_(1,26)_ < 1, NS
Y-BOCS	27.80	(3.91)	24.92	(8.21)	*F*_(1,26)_ = 1.46, *p* = 0.24
	*n*	(%)	*n*	(%)	*χ*^2^_df_
Sex (*n*/percent female)	13	(86.7)	9	(69.2)	*χ*^2^_1_ = 1.26, *p* = 0.26

^a^*M*, mean; *SD*, standard deviation; *n*, sample size; *F*, *F* statistic; *χ*^2^, chi-square statistic; *df*, degrees of freedom; *p*, *p*-value; NS, non-significant; Y-BOCS, Yale-Brown Obsessive-Compulsive Scale; (†) one participant did not provide consent for their demographic and Y-BOCS data to be shown. *Log_10_(*x* + 1) transformed Y-BOCS scores. These analyses were also conducted without the two participants excluded from the RHI contamination and habituation analyses (described above); the results remained unaltered: Age (*F*_(1,24)_ < 1, NS), Y-BOCS (*F*_(1,24)_ = 1.32, *p* = 0.26), and Sex (*χ*^2^_1_ < 1, NS).

### Multisensory Integration in OCD

RHI survival rate: all participants in the experimental condition (*n* = 16) reported a robust RHI effect; except one participant who did not provide an illusion onset, but rated the illusion as 5 out of 20 in intensity, which suggested he had a diminished RHI (based on our previous cutoff where an intensity rating of less than 3 out of 20 indicates no illusion; see Jalal et al., 2015). Surprisingly, all patients in the control condition (*n* = 13) also reported the RHI, except one who scored 2 out of 20 in intensity (another participant had a borderline illusion with an intensity rating of 5). Thus, the presence of the RHI did not differ in the two conditions ($\chi_{1}^{2}$ = 1.27, *p* = 0.26).

**Figure 4:**
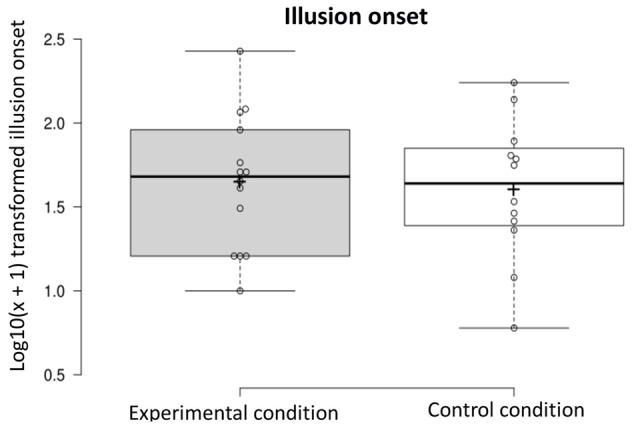
Log_10_(*x* + 1) transformed illusion onset in the experimental and control condition.

Illusion onset: on average, participants in the experimental condition reported experiencing the illusion after 65.50 s (*SD* = 68.16) vs. 57.42 s (*SD* = 51.16) in the control condition (experimental *n* = 14, control *n* = 12). A one-way ANOVA was conducted on the illusion onset dependent variable [i.e., log_10_(*x* + 1) transformed scores] to compare ratings in the experimental condition and control condition. The onset of the illusion did not differ in the two conditions (*F*_(1,24)_ < 1, NS; see Figure 4).

**Figure 5:**
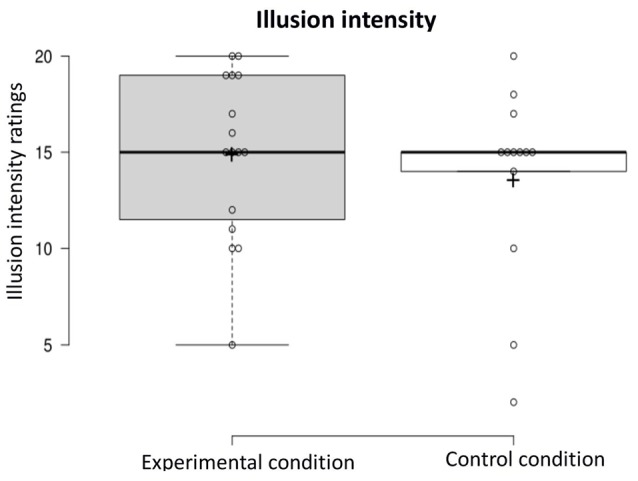
Illusion intensity in the experimental and control condition.

Illusion intensity: a one-way ANOVA was conducted on the illusion intensity-dependent variable to compare ratings in the experimental condition and control condition (experimental *n* = 16, control *n* = 13). The intensity of the illusion did not differ in the two conditions (*F*_(1,27)_ < 1, NS; see Figure 5).

OCD symptoms and RHI onset and intensity: an exploratory Pearson’s Correlation Test showed that OCD symptom severity was not associated with how soon participants experienced the RHI [i.e., log_10_(*x* + 1) transformed Y-BOCS and onset scores; *r*_11_ = −0.16, *p* = 0.61, two-tailed], in the experimental condition; similarly, such symptom severity was not associated with the strength of the illusion (*r*_13_ = 0.12, *p* = 0.67, two-tailed). However, in the control condition, while OCD symptom severity was not associated with the illusion onset (*r*_10_ = 0.15, *p* = 0.64, two-tailed), Y-BOCS scores inversely correlated with the intensity of the illusion (*r*_11_ = −0.73, *p* = 0.004, two-tailed).

### RHI Contamination (“Fake Hand Exposure”)

**Figure 6:**
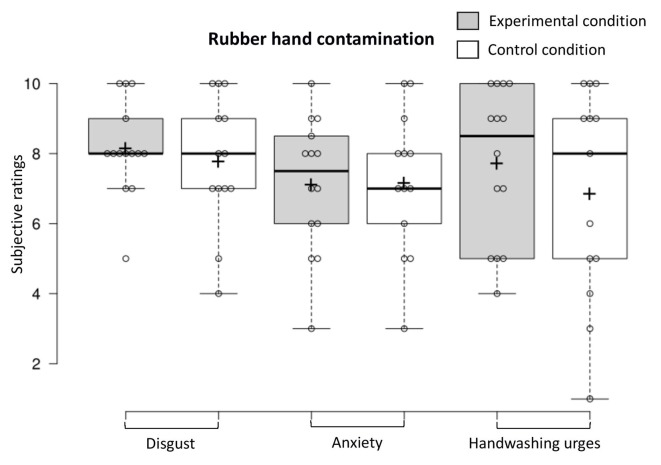
Contamination sensations ratings in the experimental and control condition during the rubber hand contamination procedure.

To examine contamination sensations when the fake hand was contaminated, we conducted a one-way MANOVA on the dependent variables (experimental *n* = 14, control *n* = 13). Contamination sensations (disgust, anxiety, and handwashing urges) did not differ in the two conditions when the fake hand was contaminated (*F*_(3,23)_ < 1, NS, Benjamini–Hochberg corrected; see Figure 6). The proportion of participants in the experimental condition and control condition who exhibited a facial expression of disgust when the fake hand was contaminated did not differ (experimental *n* = 14, control *n* = 13; $\chi_{1}^{2}$ < 1, NS, Benjamini–Hochberg corrected).

### RHI Habituation

**Figure 7:**
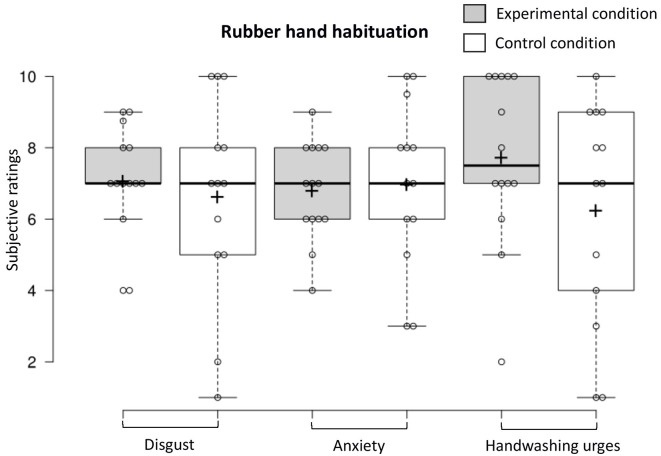
Contamination sensations ratings in the experimental and control condition during the rubber hand habituation procedure.

To examine habituation 5 min after the fake hand was contaminated, we conducted a one-way MANOVA (experimental *n* = 14, control *n* = 13) that revealed that contamination sensations (disgust, anxiety, and handwashing urges) did not differ in the two conditions (*F*_(3,23)_ = 1.22, *p* = 0.32, Benjamini–Hochberg corrected; see Figure 7). The proportion of participants who exhibited a facial expression of disgust was higher in the experimental condition vs. the control condition (experimental *n* = 13, control *n* = 13; 64.7% vs. 35.3%; $\chi_{1}^{2}$ = 4.25, *p* = 0.04, Benjamini–Hochberg corrected).

### *In vivo* Exposure Habituation

**Figure 8:**
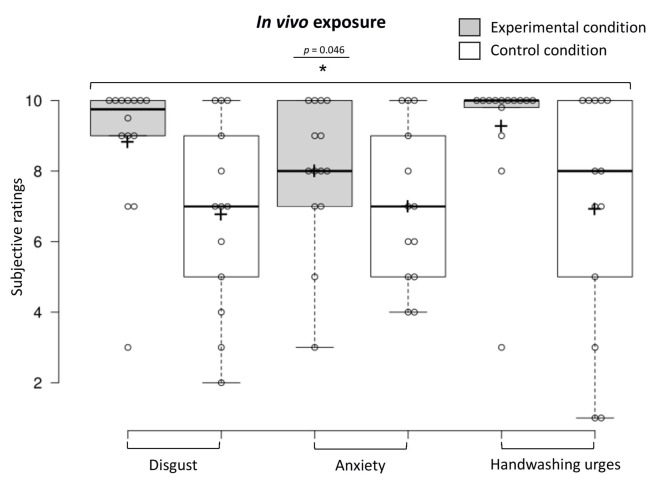
Contamination sensations ratings in the experimental and control condition during the *in vivo* exposure procedure.

To examine *in vivo* exposure habituation immediately upon discontinuing the stimulation of the real and rubber hand, we conducted a one-way MANOVA (experimental *n* = 14, control *n* = 13), showing that participants in the experimental condition reported higher overall contamination sensations (disgust, anxiety, and handwashing urges) compared to those in the control condition (*F*_(3,23)_ = 3.12, *p* = 0.046, Benjamini–Hochberg corrected; see Figure 8). The MANOVA was followed up with a discriminant function analysis that revealed one discriminant function, which significantly differentiated the experimental and control condition (Wilks’ lambda *λ* = 0.71, $\chi_{3}^{2}$ = 8.02, *p* = 0.046). A canonical correlation of 0.54 showed that the model explained 29.2% of the variation in the condition variable. The discriminant function analysis revealed that disgust ratings had the highest standardized canonical discriminant function coefficient (*β* = 2.40) indicating the greatest contribution to the model (i.e., the best discriminator between the two conditions), followed by anxiety (*β* = −1.80) and then washing urge ratings (*β* = −0.04).

### Dummy Exposure vs. *in vivo* Exposure

In an exploratory analysis, to compare contamination sensations during dummy exposure vs. *in vivo* exposure, we conducted two repeated measures one-way MANOVAs (experimental *n* = 14, control *n* = 13), showing that while *in vivo* exposure provoked more intense responses than dummy exposure in the experimental condition (*F*_(3,11)_ = 3.92, *p* = 0.04), this was not the case in the control condition (*F*_(3,10)_ < 1, NS; residuals showed moderate deviation from normality but were not improved with a log or square-root transformation and were thus analyzed with those caveats). Follow-up one-way ANOVAs showed that in the experimental condition *in vivo* contamination triggered marginally significantly greater disgust (*F*_(1,13)_ = 3.84, *p* = 0.07) and significantly greater anxiety (*F*_(1,13)_ = 7.60, *p* = 0.02) and handwashing urges (*F*_(1,13)_ = 8.81, *p* = 0.01) than dummy exposure.

## Discussion

This study yields important new findings with clinical implications. Intriguingly, our results suggest sensory assimilation of contamination sensations into the body image *via* the RHI—that such feelings were curiously referred to an alien hand in patients with OCD. Patients undergoing synchronous stimulation did not report greater contamination sensations when the fake hand was initially contaminated relative to asynchronous stroking. But contrary to expectations, they did so after the dummy had been contaminated for 5 min, as assessed *via* disgust facial expressions (a secondary outcome) and *in vivo* exposure (upon discontinuing the illusion). We also found that patients failed to reject the illusion during the “gold standard” control condition. To our surprise, synchronous and asynchronous stroking induced an equally vivid and fast-emerging illusion, which helps explain why both conditions initially (5 min after initiating tactile stimulation) provoked contamination reactions of equal magnitude. This study is the first to suggest heightened malleability of body image in OCD. Collectively, these results argue against a sharply localized (“hierarchical”) approach to brain function and illustrate dynamic intersensory interactions and plasticity of brain modules (“holistic mediation”).

Our findings stress the importance of the temporal dimensions of the RHI, and crucially, how these can be perturbed by psychopathology. As noted, our chosen duration of tactile stimulation (i.e., 5 min) prior to dummy contamination was insufficient to initially differentiate the synchronous and asynchronous conditions in patients with severe OCD. By comparison, we have previously shown that 5 min of tactile stimulation differentiates the RHI and the control condition in healthy individuals (Jalal et al., 2015). In the current study, indeed, as both methods of stroking triggered an equally intense illusion at this time point, one would expect them to provoke comparable contamination reactions. But over time, these results suggest that synchronous stimulation more effectively assimilated the visibly contaminated rubber hand into the body image (than asynchronous stroking)—accounting for the relative rise in contamination sensations. Although we did not explicitly assess illusion intensity at a later stage, this provides a viable explanation for why synchronous stroking differentially impacted contamination reactions 10 min after initiating stimulation on two separate measures. As mentioned, research suggests that the RHI becomes more intense with time (i.e., duration of stimulation), as indexed on a key measure of the illusion (i.e., perceiving one’s real hand drifting towards the fake one; Tsakiris and Haggard, 2005; on the prevalence of the RHI over time and degree of proprioceptive drift, see also Botvinick and Cohen, 1998).

The formulation of the initial hypothesis that contaminating the fake hand during the RHI results in greater contamination sensations than does asynchronous stroking in OCD, *specifically* 5 min after beginning the stroking, was based on prior work in healthy volunteers (Jalal et al., 2015; see also Nitta et al., 2018). Evidently, in this study, as the RHI triggered greater contamination reactions than did the control procedure, not 5 min but instead 10 min after stroking began (consistent with the overall hypothesis, but not the timeline in which the two conditions were differentiated), our study design was unable to capture any habituation effects. Nevertheless, given the literature on ERP (e.g., Foa et al., 1983; Rachman, 2004; Abramowitz, 2006; McKay, 2006; i.e., the basis for the second hypothesis), we can safely assume that such fake hand exposure would eventually lead to habituation (i.e., causes a gradual decrease in these sensations as extinction occurs). As our exposure method proved highly potent at evoking contamination reactions (surprisingly, irrespective of stroking approach), it may be that akin to ERP, at least 30–45 min of continuous exposure is needed for habituation to occur, bearing in mind that patients vary in the rate of habituation (e.g., Simpson et al., 2010). Future research should further disentangle such habituation timeline.

That a higher proportion of patients exhibited a disgust facial expression during the RHI relative to the control condition (65% vs. 35%; i.e., 5 min after the fake hand was contaminated, 10 min after stroking began) is consistent with the key role of disgust in OCD (Ludvik et al., 2015), e.g., as a strong predictor of contamination fears (e.g., Olatunji et al., 2005; see also Deacon and Olatunji, 2007; Olatunji et al., 2007). This measure provides an objective assessment of disgust.

The results of the exploratory analysis are noteworthy. They emphasize the overall finding that synchronous stroking over time exerts selective sensitizing effects (i.e., vis-à-vis contamination reactions). But more strikingly, they imply that “fake hand exposure” during asynchronous stroking provokes contamination sensations as effectively as actual real hand exposure. This finding is highly counterintuitive. It dovetails with our related studies showing that both college students with OCD symptoms (Jalal and Ramachandran, 2017) and severe OCD patients (Jalal et al., under review) report indistinguishable levels of disgust when merely watching an experimenter contaminating his own hand and when their hand is contaminated. This research illustrates the cognitive impenetrability of contamination sensations (i.e., how such gut reactions can override logic and break down “self-other” barriers). Intriguingly, they also suggest that direct skin contamination may be unnecessary to gain the beneficial effects of exposure therapy. Contaminating proxy stimuli such as alien limbs (synthetic or biological) can potentially trigger clinically relevant contamination reactions (see also, Jalal et al., 2018).

In this study, we found an overall amplified RHI. For instance, all patients reported the illusion during synchronous stroking. In contrast, around 85% of healthy volunteers experience the effect (Jalal et al., 2015). But the finding that patients failed to reject the RHI during asynchronous stroking is more notable. It mirrors research showing that both Parkinson’s disease and schizophrenia patients exhibit heightened illusory effects during asynchronous stroking compared to healthy volunteers (Peled et al., 2000; Ding et al., 2017), and that dopamine releaser drugs ketamine and dexamphetamine enhance the RHI during both synchronous and asynchronous stimulation (Albrecht et al., 2011; Morgan et al., 2011). Taken together, these data indicate that dopamine dysregulation may boost a sense of embodiment. As noted, although the role of dopamine in OCD is admittedly complex (Fineberg et al., 2007), research has shown that dopamine antagonists can be useful in reducing OCD symptoms (as an adjunct to SSRIs; Vulink et al., 2009) and that dopamine agonists can generate OCD-like behaviors [Borcherding et al., 1990; Szechtman et al., 1998; of interest, ketamine *per se* shows affinity for dopamine D_2_ in addition to serotonin 5-HT_2_ receptors (Kapur and Seeman, 2002) both blocked by quetiapine, an antipsychotic sometimes used in the treatment of refractory OCD (Gefvert et al., 2001)].

Notably, dopamine has been linked to learning (e.g., Centonze et al., 2001; Castner and Williams, 2007) and is found in brain areas underlying the RHI (Ehrsson et al., 2004; on dopaminergic projections to the prefrontal cortex, see Goldman-Rakic et al., 1990). It could, therefore, contribute to perceptual learning processes mediating corporeal awareness and possibly account for an amplified illusion in OCD. But how does dopamine induce the RHI in the face of contradictory input (i.e., asynchronous stimulation)? One explanation is that dopamine overactivity underlies salience attribution: ascribing causal importance to salient events (e.g., Howes et al., 2015). In the asynchronous condition, the patient focuses his attention on a dummy that resembles the patient’s hand and it appears in its expected location. This attention-grabbing input violates expectations, rendering the event highly salient. As such, learning (“dopamine-encoding”) might ensue, i.e., driving the illusion of ownership (“the fake hand on the table must be mine”) even when incoming sensory information is incongruous, effectively overriding internally constructed models of reality (Albrecht et al., 2011; on Bayesian prediction error, see Fletcher and Frith, 2009). Together, these findings stress how a unified sense of self may rest on a delicate balance between top-down regulation and bottom-up processes.

Another noteworthy factor to consider is the idiosyncratic perceptual style in OCD, possibly exacerbating such dopamine-driven top-down visual processing and salience misattribution. As early as the 1960s, Shapiro described the obsessive-compulsive attentional style: a painstaking focus on minor details in a rigid manner, at the expense of the big picture—effectively “missing the forest for the trees” (Shapiro, 1965; see also Yovel et al., 2005). Research has since shown that patients with OCD indeed focus on local aspects of visual stimuli instead of holistic, organizational features (Savage et al., 1999); i.e., in line with neurocognitive models implicating frontal–striatal abnormalities in OCD (e.g., mediating cognitive inflexibility circuits; Vaghi et al., 2017; for reviews, see Menzies et al., 2008; Nakao et al., 2014) [Similar tendencies occur in students with OCD symptoms (Soref et al., 2008) and individuals with obsessive-compulsive personality disorder (Yovel et al., 2005)]. Accordingly, OCD patients in the asynchronous condition when asked to focus on the fake hand did so in an intensely focused and inflexible manner, conceivably causing them to ignore the overall conflicting sensory information, i.e., leading to global degradation in multisensory integration and overreliance on the salient visual input. This explanation dovetails with the finding that patients with the etiologically related OCD spectrum (“fronto-stratial”) disorder BDD (Grace et al., 2017) display proprioceptive drift bias towards the fake hand during both synchronous and asynchronous stimulation. Unsurprisingly, patients with BDD, like those with OCD, focus on perceptual details at the cost of global, holistic processing (Deckersbach et al., 2000), fittingly evoked as an explanation for such unusual proprioceptive drift bias in BDD (Kaplan et al., 2014).

Counterintuitively, Y-BOCS scores inversely correlated with the intensity of the illusion but only during asynchronous stimulation. One explanation for this is that top-down attention, possibly driving the illusion during asynchronous stroking (*via* salience misattribution), was perturbed by anxiety states in the most severe patients. Indeed, anxiety decreases attentional control (Eysenck et al., 2007) and is unsurprisingly associated with OCD symptoms (e.g., Foa et al., 1998). Anxiety overall may, therefore, have interfered with perceptual learning effects of dopamine (caused “general blunting”), which might explain why OCD severity (irrespective of condition) did not intensify the illusion.

The primary aim of this study was to explore the therapeutic potential of the RHI. Our findings may pave the way for a novel therapeutic technique for OCD (see also Jalal et al., 2015). Practically (e.g., based on the current results), such an approach might entail 10 min of tactile stimulation, coupled with at least 5 min of continuous dummy contamination (as outlined in the “Materials and Methods” section). The procedure should be repeated (e.g., 3–4 times) until habituation occurs; for severe patients, possibly starting with asynchronous stroking followed by synchronous for a more immersive experience [Analogously, a session of ERP typically lasts around 90 min (van der Heiden et al., 2016)].

This method we have introduced may offer a tolerable alternative to ERP, with potential to trigger clinically relevant contamination reactions. Crucially, unlike ERP, it does not require patients to touch highly aversive “contaminants.” As such, it is conceivable that patients who are reluctant to engage in ERP due to fear of direct skin exposure (i.e., too frightened to confront contaminants head-on) would be more accepting of this approach. Also, as noted, it might be useful during the initial stages of exposure to help desensitize patients such that they are willing to eventually undertake ERP.

Because the RHI itself is engaging—fittingly labeled a “mind-blowing party trick” (Lawton, 2009)—our method might appeal to a younger audience. During pilot work, volunteers often express astonishment (sometimes even slight giggling) at the uncanny sensation of touch arising from an obvious fake hand. This element of amusement (positive affect) could establish a frame for a less fearful outlook on exposure, i.e., create nonthreatening re-association to bodily contamination. All in all, this simple, immersive, and cost-effective intervention might result in higher treatment uptake and lower dropout and facilitate early intervention. It is eminently suitable for poorly resourced and emergency settings, including low-income and developing countries with minimal access to high-tech solutions like virtual reality.

Although this is the first investigation to explore the RHI in OCD, our assessment of multisensory integration *per se* (a secondary study-aim) was limited in several ways. For instance, we did not take into account the impact of comorbid psychiatric conditions that may have affected these results. Indeed, as noted, psychiatric disorders have been shown to differentially influence self-referential processing. Ideally, future studies should explore corporeal awareness in OCD using large samples of unmedicated patients without comorbidities (albeit severe OCD patients without comorbidities are rare). This is particularly important because of the role of dopamine as a modulator of multisensory integration, with dopaminergic agents sometimes used as an adjunct to SSRIs in the treatment of OCD.

In this study, we assessed the RHI with a subjective intensity measure in addition to the onset rating. Although a single-item intensity measure is limited compared to embodiment questionnaires (Botvinick and Cohen, 1998; Peled et al., 2000), it has proven reliable in studies by us (Jalal et al., 2015; see also Armel and Ramachandran, 2003) and others (Lev-Ari et al., 2015; Lev-Ari and Hirschmann, 2016; Nitta et al., 2018). Future research examining multisensory integration in OCD should include additional measures such as questionnaires and the objective “proprioceptive drift” test (e.g., Botvinick and Cohen, 1998; Tsakiris and Haggard, 2005; Longo et al., 2008; Marotta et al., 2016). Although we have provided evidence vis-à-vis multisensory processing in healthy volunteers from our previous research (i.e., serving as a comparison to the current findings; Jalal et al., 2015), future studies should include a healthy control group; that we did not include one constitutes a limitation.

Consistent with Botvinick and Cohen (1998) seminal investigation, in the current study, the asynchronous control condition was a between-subject factor. Using the same sample across conditions would have been optimal for assessing self-referential processing (i.e., due to reduced variance arising from individual differences, e.g., sensory suggestibility, see Marotta et al., 2016). However, the key aim of this study was to explore the clinical potential of the RHI in OCD (specifically in severe patients undergoing IRT, often refractory to treatment in other settings). As such, our design ensured that patients were not subjected to high stress by being exposed to aversive contaminants twice (while present at our treatment center for a limited period), and, importantly also, prevented carry-over effects from the exposure procedures (e.g., habituation). Indeed, with our main clinical objective in mind, our sample was suitable for the following reasons: (1) comorbid and secondary diagnoses are common in OCD patients, who often tend to be medicated. Thus, our sample was typical of this patient population; (2) severe OCD patients may be the most fearful of ERP (i.e., entailing direct contamination) and thus generally the most in need of gentler, more tolerable treatments.

Future double-blind placebo-controlled trials should directly compare our proposed “dummy contamination” procedure to ERP. Finally, “multisensory stimulation therapy” lends itself to other applications in psychiatry (Jalal et al., 2015)—like treating “needle phobia.” Conducting realistic exposures in this population is challenging: repeated needle injections into a real arm could result in punctured veins. Using a fake hand during the RHI, instead, may provide a clever and convenient alternative.

## Data Availability Statement

The raw data will be made available by the authors to any qualified researcher upon request.

## Ethics Statement

The studies involving human participants were reviewed and approved by Harvard University. The patients/participants provided their written informed consent to participate in this study.

## Author Contributions

BJ, RM, JE, and VR designed the study. BJ performed the data analysis and wrote the manuscript. RM contributed to data interpretation and edited the manuscript. JE and SP recruited and tested patients, and edited the manuscript. VR edited the manuscript. All authors read and approved the submitted version.

## Conflict of Interest

The authors declare that the research was conducted in the absence of any commercial or financial relationships that could be construed as a potential conflict of interest.
